# 76215 Implementation of Proteomics as a Diagnostic tool for Nontuberculous mycobacteria (NTM) Infection

**DOI:** 10.1017/cts.2021.759

**Published:** 2021-03-30

**Authors:** Nicole Lapinel, Jessie Guidry, Mary Varkey, Manish Rijal, Arnold Zea, Juzar Ali

**Affiliations:** Louisiana State University Health Sciences Center

## Abstract

ABSTRACT IMPACT: Implementation of proteomics as a diagnostic tool for Nontuberculous mycobacteria (NTM) infection can provide a more accurate, efficient and cost-effective means for effectively diagnosing disease and enacting timely management decisions which can revolutionize patient care. OBJECTIVES/GOALS: Proteomic analysis is a proven diagnostic modality enabling rapid identification of microorganisms. We sought to apply proteomics to detect proteins unique to the most clinically relevant NTM. We then determined whether these unique proteomes could be used to successfully identify NTM species from in vitro cocktail preparations. METHODS/STUDY POPULATION: NTM reference strains for M. avium, m. intracellulare, m.chimaera, m. abscessus abscessus, m. abscessus massiliense and m. abscessus boletti were cultured in vitro and subjected to proteomic analysis using Liquid Chromatography tandem-Mass Spectrometry (LCMS). Tandem Mass Tag (TMT) data acquisition utilized an MS3 approach for data collection using Proteome Discoverer 2.4.

A comparative analysis of the proteome of each of these six species was performed quantitatively using LCMS. The process was repeated for three technical replicates and analyzed using the SEQUEST algorithm. Only high scoring peptides were considered utilizing a false discovery rate (FDR) of 1%. Once species-specific proteins were identified, we validated detection in individual and mixed samples of the six reference strains. RESULTS/ANTICIPATED RESULTS: The proteomic profiling of the six NTM reference strains successfully demonstrated proteins unique to each of the MAC species and MABC subspecies. Proteomic MAC species analysis produced between 327 to 2,540 unique peptides for each of the 3 species. MABC proteomic analysis identified between 17-74 unique peptides for each of the 3 subspecies. Fifteen different mixed preparations of MAC and MABC were then subjected to LCMS analysis and compared against the proteome profiles already curated for the six strains. We accurately identified at least one NTM in the majority of the samples (10/15). In three samples (3/15), the NTM was not correctly identified; in two of the samples (2/15) we were unable to determine the identity of NTM within the preparation. Further database curation will be performed to hone these results. DISCUSSION/SIGNIFICANCE OF FINDINGS: Proteomic analysis of in vitro reference strains successfully demonstrated protein fingerprints specific to six common disease-causing strains of NTM. Such findings can be used to evaluate clinical samples enabling more efficient diagnostic specificity. Further research will focus on identification of NTM in sputum samples of infected patients.

